# Determinants of Antenatal Care Service Satisfaction among Women in Ethiopia: A Systematic Review and Meta-Analysis

**DOI:** 10.1155/2022/9527576

**Published:** 2022-03-04

**Authors:** Kenbon Seyoum

**Affiliations:** Department of Midwifery, Madda Walabu University Goba Referral Hospital, Goba, Ethiopia

## Abstract

**Background:**

Antenatal care service satisfaction is a measure of the degree to which a woman seeking care is happy with the antenatal care service provided to her. Thus, this systematic review and meta-analysis aims to identify factors that determine antenatal care service satisfaction among women in Ethiopia.

**Methods:**

PubMed, Hinari, and Google Scholar were systematically searched for eligible studies. In addition, national university digital libraries were also searched. The Joanna Briggs Institute's (JBI) critical appraisal tools were used to assess the quality of the included articles. The Cochrane Q-statistics and I2 tests were used to assess heterogeneity among the included studies. Publication bias was assessed using Egger's test. Joanna Briggs Institute Critical Appraisal Checklist for Analytical Cross-Sectional Studies. The extracted data were analyzed using STATA version 14 software and the results were presented using the forest plot.

**Results:**

Of the 274 articles identified through the systematic search of the literature, 13 studies fulfilling the inclusion criteria were included in this meta-analysis. First antenatal care visit (AOR: 0.62 and 95% CI: 0.40, 0.96), women waited <60 min (AOR: 1.87 and 95% CI: 1.40–2.50), women whose privacy was maintained (AOR: 3.91 and 95% CI: 1.97–7.77), women treated respectfully (AOR: 5.07 and 95% CI: 2.34–10.96), and unplanned pregnancies (AOR = 0.28 and 95% CI: 0.10–0.77) were significantly associated with antenatal care service satisfaction.

**Conclusion:**

The study assessed the determinants of antenatal care service satisfaction in Ethiopia. First antenatal care visit, waiting time (<60 min) to see the care provider, maintenance of privacy, respectful treatment, and pregnancy unplanned were found to be determinants of antenatal care service satisfaction. Counseling a woman to comply with a minimum required antenatal care visits and compassionate and respectful maternity care will increase maternal satisfaction with the antenatal care services.

## 1. Introduction

Patient satisfaction is a measure of the degree to which a health care seeking client or patient is happy with the care given by health care providers. It is important to define the success of health care facility [1]. Dissatisfaction of health care services by the mother will hinder her from receiving modern health care [2]. Poor quality of care contributes to maternal morbidity and mortality [3]. Maternity is the state or quality of being a mother [4]. Maternal care is care given to a woman during pregnancy, childbirth, and the postpartum period [5]. It is the care that enhances the likelihood for the pregnant woman to regularly go for check-ups to assess risks, to screen for and treat antenatal conditions, to enhance the effective management of obstetric emergencies during the childbirth process, and to encourage postpartum care that is important for detecting and treating postpartum complications [6]. The use of skilled maternity care can be deterred by the fear of disrespecting and mistreatment in maternity care providing health facilities [7].

Patient satisfaction is an indirect indicator of the quality of health care services [8]. It is also an important indicator of accessibility and quality of care which, in turn, shows the performance of the health care system [9]. Deficient health service leads to patient dissatisfaction and dissatisfied patients do not patronize the same institution, which has economic and organizational quality implications [10].

Antenatal care is care provided to pregnant women by skilled health care professionals to ensure the best health conditions for both the baby and mothers during pregnancy. Risk prevention, identification, and management of pregnancy-related or coexisting diseases, and health education and promotion are the components of antenatal care [11]. The World Health Organization recommends quality antenatal care for all women to ensure positive pregnancy outcomes [12]. The quality of antenatal care is important and settings with low resources, shortages in essential equipment or medicines, and lack of skilled staff struggle to offer quality antenatal care [13].

Although a number of studies have identified determinants of antenatal care service satisfaction in Ethiopia, these tend to be limited to certain areas and present findings which are highly variable, inconsistent, and nonrepresentative. In this regard, the absence of a nation-wide study was identified as a significant gap. Thus, this systematic review and meta-analysis are meant to summarize the determinants of antenatal care service satisfaction among women in Ethiopia. The findings from this study will help antenatal care providers, policymakers, and concerned bodies to know antenatal care dissatisfying factors, and modifying or intervening them.

## 2. Materials and Methods

### 2.1. Search Strategy

The study protocol was registered in the International Prospective Register of Systematic Reviews (PROSPERO), the University of York Centre for Reviews and Dissemination (ID number: (CRD42019137013). This review and meta-analysis were conducted according to the guideline of Preferred Reporting Items for Systematic reviews and Meta-Analysis (PRISMA) [14]. Protocol was published on PROSPERO and can be found using the registration number provided above.

### 2.2. Eligibility Criteria

#### 2.2.1. Inclusion Criteria

*(1) Setting*. Only quantitative studies carried out in health institutions in Ethiopia were included in this systematic review and meta-analysis.

*(2) Language*. Only articles published in English were retrieved for review. No articles published in another language were found.

*(3) Publication Condition*. Both published and unpublished articles were considered for this review.

 
*Intervention(s)/exposure(*s): All pregnant women attending antenatal care.
 
*Outcome*: The primary outcome is the determinants of ANC service satisfaction among woman in Ethiopia. It is defined as the degree to which an antenatal care seeking woman is happy with the antenatal care given by health care providers, which was measured by different sociodemographic and care provider factors.

#### 2.2.2. Exclusion Criteria

Studies for which we are unable to get the necessary details after contacting the authors were excluded. Studies on intranatal care, case series, and case studies were also excluded.

### 2.3. Information Sources and Search Strategy

The following databases were searched to find potentially relevant articles: PubMed, Hinari, and Google Scholar. No date limit was applied. National university digital libraries, such as the electronic library of Addis Ababa University, were searched to include gray literature. Hand searches of the reference lists of all included studies were also conducted. Identified articles were directly transferred to endnote citation manager for inclusion.

Search terms such as “magnitude”, “prevalence”, “determinants,” “associated factors,” “antenatal care,” “prenatal care,” and “satisfaction” were used. The Boolean operators (OR and AND) were used to combine search terms. Examples of a searching strategy fit for all the databases searched are available in supporting information (Additional file 1).

The following procedures are guided this systematic review. First, the electronic database search results were imported into the reference management software (Endnote™) and all duplicates were removed. In the second step, all articles were screened by their title, abstract, and full text for eligibility against the predefined inclusion and exclusion criteria. Third, a full document manuscript review was conducted and studies were removed through the predefined exclusion criteria. Finally, the included articles were evaluated based on the Joanna Brigg's Institute (JBI) quality assessment tool [15, 16].

### 2.4. Data Selection and Extraction

The articles for this study were selected guided by a PRISMA flow diagram. Data charting and screening process was performed independently (KS) based on eligibility criteria by using Microsoft Excel™. This was conducted after extracting the titles and abstracts of the eligible studies and after removing duplicates. The retrieved articles were uploaded to endnote software citation manager and duplicates were removed. The data extraction form included the name of the author, year of publication, regions where the study was conducted, sample size, response rate, setting, and type of study design. The log odds ratios with 95% confidence intervals for the included variables were extracted in a binary format. The extracted data were imported to STATA version 14 for data analysis. The data collection and checking was conducted by a single author (KS) and two corresponding authors of the included studies were contacted twice through e-mail provided on their published papers.

### 2.5. Risk of Bias

Risk of bias was assessed using JBI Critical Appraisal Checklist for Analytical Cross-Sectional Studies [15]. The checklist has 8 parameters: (1) criteria for inclusion in the sample clearly defined; (2) study subjects and the setting described in detail; (3) exposure measured in a valid and reliable way; (4) objective, standard criteria used for the measurement of the condition; (5) confounding factors identified, (6) strategies to deal with confounding factors stated; (7) the outcomes measured in a valid and reliable way; and (8) was appropriate statistical analysis used? All articles assessed had a high score and all of them were included in the study (additional file 2). The risk of bias assessment was conducted independently by the researcher.

### 2.6. Data Synthesis

The odds ratio for determinant data were computed by pooling odds ratio reported in the original studies and the standard error (SE) for the natural logarithm of odds ratios (ln OR) were calculated using formula SE (ln OR) =  $\sqrt{\left(1/a\right)+\left(1/b\right)+\left(1/c\right)+\left(1/d\right)}$. The pooled odds ratio with their 95% CI were presented using forest plot. Both the random and fixed effect model was used to determine determinants of ANC service satisfaction. Heterogeneity of the reported pooled odds ratio was assessed by computing Cochrane Q-statistic and *I*^2^statics. *I*^2^ test statistics result of 25%, 50%, and 75% was declared as low, moderate, and high heterogeneity, respectively [17]. STATA version 14 software (StataCorp LP.2015, College Station, TX : USA) were used for all statistical analyses.

### 2.7. Publication Bias

Egger's test was used to assess publication bias. A *P*-value of less than 0.05 was used to declare the publication bias.

### 2.8. Sensitivity Analysis

A leave-one-out Sensitivity analysis using a random-effects model was performed to assess the influence of a single study on the overall pooled estimate. The result of sensitivity analysis is given in an additional file 3.

### 2.9. Operational definition

 
**Unplanned pregnancies** occur when no children or extra children are not needed or when the pregnancy occurs earlier than desired.
 
**Respect** refers to what pregnant women think about their treatment with dignity and compassion.
 
**Satisfaction** shows how happy an antenatal care seeking woman is with the care provided by the antenatal care provider.
 
**Privacy** in this study refers to absence of another person in the antenatal care room during providing antenatal care and consultation service to a woman.

## 3. Results

### 3.1. Selection of Sources of Evidence

**Figure 1:**
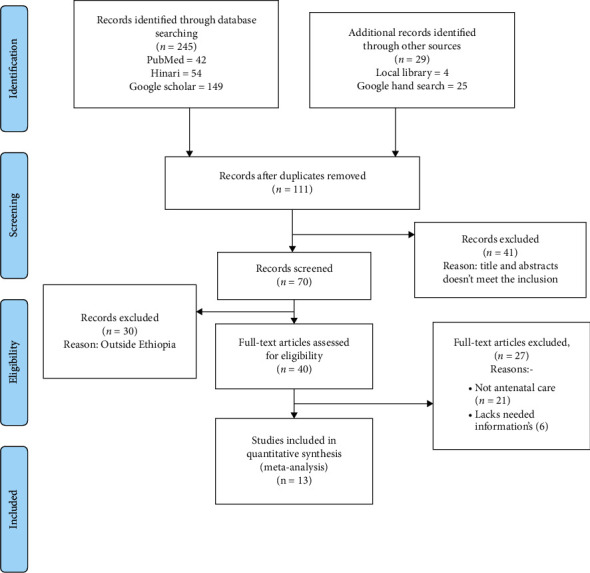
PRISMA flow diagram of study selection for systematic review and meta-analysis of determinants of antenatal care service satisfaction among women in Ethiopia.

A total of 274 articles were retrieved during our primary search. Of these, 163 articles were excluded due to duplication. The titles and abstracts of the remaining 111 articles have been screened and 41 articles that do not meet the inclusion criteria were removed. The remaining 70 articles were screened in full, and 30 articles that were found to be outside the study area were removed. The full text of the remaining 40 articles were also assessed in detail against the eligibility criteria, and 27 articles were excluded as they did not have any important information (no relevant information and not prenatal care). Finally, 13 articles that meet the inclusion criteria are left for final analysis (Figure 1).

### 3.2. Characteristics of the Included Studies

Thirteen studies are included in this review. All of them are cross-sectional by design. Six of the included studies were from Southern Nation Nationalities And Peoples Region (SNNPR) [18–23], four from Oromia [24–27], one from Amhara region [28], one from Harari [29], and one from Addis Ababa city [30]. Regarding the response rate, all studies had a good response rate (>88%) (Table 1).

### 3.3. Determinants of Antenatal Care Service Satisfaction

#### 3.3.1. Association between Maternal Age and Antenatal Care Service Satisfaction

**Figure 2:**
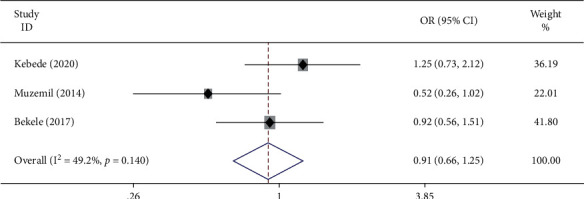
Forest plot exhibiting the pooled odds ratio of the association between maternal age and antenatal care service satisfaction among women in Ethiopia.

The association between maternal age and antenatal care service satisfaction was examined based on the findings from three studies [21, 25, 30]. The pooled odds ratio (AOR: 0.91, 95% CI: 0.66–1.25) showed that antenatal care service satisfaction is not affected by the age of mother. The studies showed moderate heterogeneity (*I*^2^ = 49.2% and *P* = 0.140) (Figure 2). Hence, a fixed effects model was employed to do the final analysis.

#### 3.3.2. Association between Maternal Education and Antenatal Care Service Satisfaction

**Figure 3:**
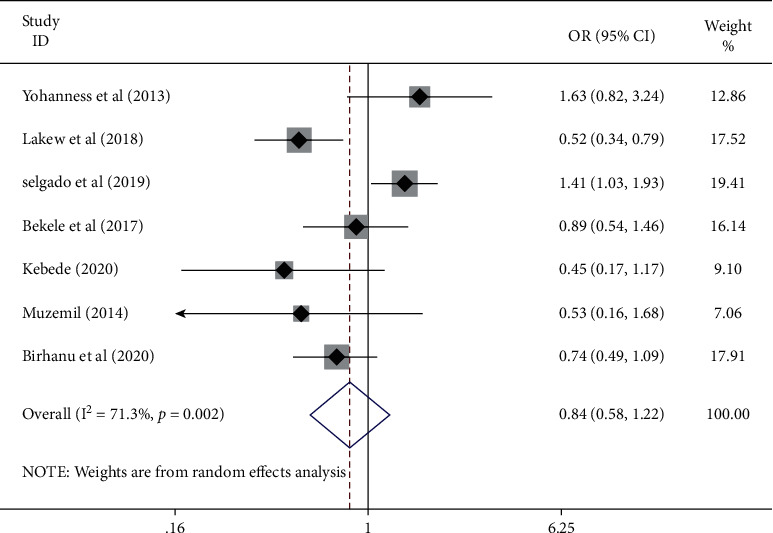
The pooled odds ratio of the association between maternal education and antenatal care service satisfaction among women in Ethiopia.

Seven studies [19, 21, 23–25, 29, 30] reported the association between women's education and antenatal care service satisfaction. The pooled results of these studies showed that antenatal care service satisfaction is not affected by women's educational status. The pooled odds ratio of the study is (OR: 0.84, 95% CI: 0.58–1.22). The study showed a heterogeneity of 74.55 and *P* ≤ 0.01. Egger's test revealed the absence of publication bias (*P* = 0.41) (Figure 3).

#### 3.3.3. Association between Number of Antenatal Care Visit and Antenatal Care Service Satisfaction

**Figure 4:**
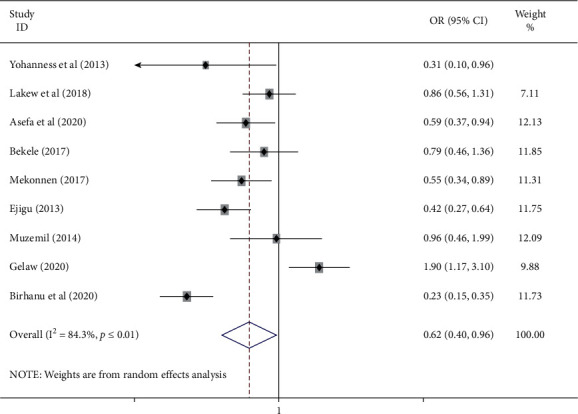
Forest plot showing the pooled odds ratio of the association of number of antenatal care visit and antenatal care service satisfaction among women in Ethiopia.

The meta-analysis to test the association between antenatal care service satisfaction and number of antenatal care visits was based on the result of nine studies [19, 20, 22, 23, 25, 26, 28–30]. The pooled odds ratio (AOR = 0.62, 95% CI: 0.40, 0.96) showed that pregnant women having first ANC were 38% less likely to be satisfied than women having more than one visit. The I^2^ test showed the existence of high heterogeneity (I2 = 84.3%, *P* ≤ 0.01). Egger's test revealed the absence of publication bias with a *P*-value of 0.85 (Figure 4).

#### 3.3.4. Association of Waiting Time and Antenatal Care Service Satisfaction

**Figure 5:**
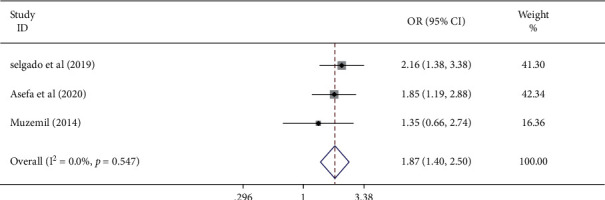
Forest plot displaying the pooled odds ratio of the association of waiting time and antenatal care service satisfaction among women in Ethiopia.

This study assessed the association between waiting time to see an antenatal care providers and antenatal care service satisfaction using three studies [24, 26, 30]. The results of this meta-analysis showed that a waiting time of <60 minutes was positively associated with antenatal care service satisfaction. The pooled odds ratio indicated that women waiting <60 minutes had 1.87 times (AOR: 1.87, 95%; CI: 1.40–2.50) higher odds of ANC satisfaction compared to their counterparts. The included studies showed an absence of heterogeneity (*I*^2^ = 0.0% and *P* < 0.547) (Figure 5).

#### 3.3.5. Association of Privacy and Antenatal Care Service Satisfaction

**Figure 6:**
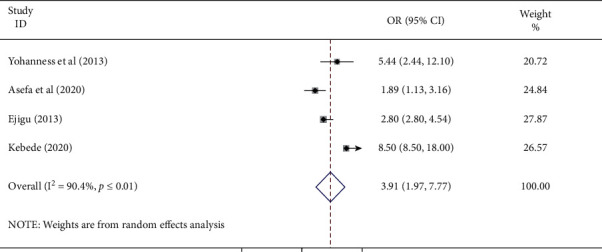
The pooled odds ratio of the association of maternal privacy and antenatal care service satisfaction among women in Ethiopia.

This study measured the association between maintenance of privacy and antenatal care service satisfaction using four studies [21, 23, 26, 28]. And showed that maintenance of privacy was positively associated with antenatal care service satisfaction. The pooled odds ratio displayed that women whose privacy was maintained had 3.91 times (AOR: 3.91, 95%; CI: 1.97–7.77) higher odds of ANC satisfaction compared to those whose their privacy were not maintained. The included studies showed high of heterogeneity (*I*^2^ = 90.4% and *P* ≤ 0.01). Hence, a random effect meta-analysis was employed (Figure 6).

#### 3.3.6. Association of respect and Antenatal Care Service Satisfaction

**Figure 7:**
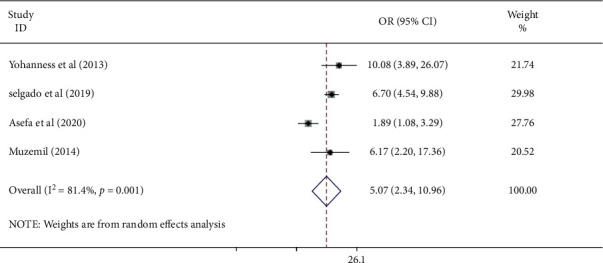
Forest plot demonstrating the pooled odds ratio of the association of respectful treatment and antenatal care service satisfaction among women in Ethiopia.

This study also assessed the association between respectful treatment of women and antenatal care service satisfaction using four studies [23, 24, 26, 30]. The pooled results of this meta-analysis exhibited that respectful treatment of clients was positively associated with antenatal care service satisfaction. The pooled odds ratio showed that women who were treated respectfully had 5.07 times (AOR: 5.07, 95%; CI: 2.34–10.96) higher odds of ANC satisfaction compared to their counterparts. The included studies showed high of heterogeneity (*I*^2^ = 81.4% and *P* = 0.001). Hence, a random effect meta-analysis was employed (Figure 7).

#### 3.3.7. Association of Place of Residence and Antenatal Care Service Satisfaction

**Figure 8:**
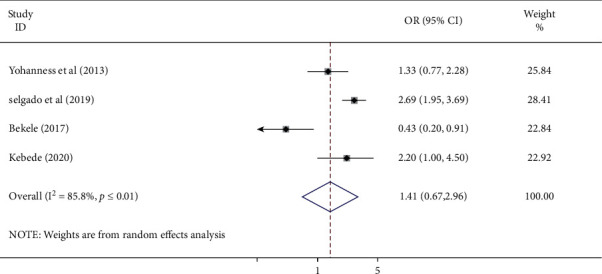
Forest plot showing the pooled odds ratio of the association of maternal place of residence and antenatal care service satisfaction among women in Ethiopia.

This meta-analysis evaluated the associations between place of residence and antenatal care service satisfaction based on four studies [21, 23–25]. The pooled results of the odds ratio revealed the absence of an association between maternal place of residence and antenatal care service satisfaction (AOR: 1.41, 95%; CI: 0.67–2.96). The studies displayed high heterogeneity (*I*^2^ = 85.8% and *P* ≤ 0.01). Hence, a random effect meta-analysis was employed (Figure 8).

#### 3.3.8. Association of Type of Pregnancy and Antenatal Care Service Satisfaction

**Figure 9:**
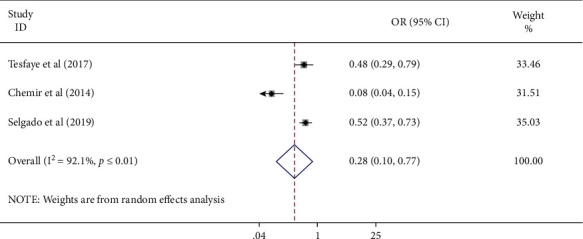
Forest plot displaying the association of unplanned pregnancy with antenatal care service satisfaction among women in Ethiopia.

The association between type of pregnancy and ANC service satisfaction was examined using three studies [18, 24, 27]. The pooled odds ratio exhibited that women who had unplanned pregnancies had 72% time (AOR = 0.28, 95 CI: 0.10–0.77) lower odds of ANC service satisfaction compared to women who had planned pregnancy. The studies demonstrated high heterogeneity (*I*^2^ = 92.1% and *P* ≤ 0.01). Hence, a random effect meta-analysis was employed (Figure 9).

## 4. Discussion

This meta-analysis assessed factors determining antenatal care service satisfaction among women in Ethiopia. It demonstrates that frequencies of antenatal care visits, waiting time, privacy maintained, respectful treatment of mothers, and pregnancy unplanned were found to determine maternal satisfaction with antenatal care services.

This study found that antenatal care service satisfaction among women having one visit is lower than women having more than one. The awareness of the importance of ANC services may increase with a repeated visits. The service, advice, and building of the relationship between the client and antenatal care providers as the number of visits increases may increase satisfaction with the antenatal care services. Repeated visits may offer women chance to ask her concerns and increase awareness of its importance. Development of a positive relationships between providers and client, increasing client needs, and effective response to this need by the healthcare professional may also increase maternal satisfaction with antenatal care service [19, 29].

This study also indicated that ANC service satisfaction is also influenced by the length of time a woman spent to see a health care providers. In this study, women who waited for <60 minutes to see an antenatal care provider had 1.87 times higher odds of ANC satisfaction compared to women who waited more. The woman may think her time is wasted and she left disregarded when she waited for a long time. This is supported by studies from Myanmar [31], Ghana [32], Kenya, and Namibia [33], which concluded that long waiting time to see the doctor was among the services that reduces client satisfaction. The study also indicated that efficiency of services refers to the promptness of the care given to patients, and short waiting time to see the health care provider was linked with high satisfaction, where the longer waiting time linked with low level of client satisfactions [34].

This meta-analysis also identified that maintenance of privacy and antenatal care service satisfaction has a positive relationship. Women whose privacy is maintained may get the freedom to discuss about her concerns with both a male and female health care providers. However, the presence of another person in the ANC room may make a woman feel unsecured during consultation [26, 28].

This study revealed that respectful treatment of women has a positive relationship with antenatal care service satisfaction. In this study, women who were treated respectfully had 5.07 times higher odds of ANC satisfaction compared to their counterparts. The probable explanation is women who feel respected (e.g., greeted warmly and counselled) may think the service/care is good and that may make her to continue to seek health care in the future also. This is supported by a study conducted in Malawi which stated that women will not seek service if she is disrespected [35].

This study showed a negative association between antenatal care service satisfaction and unplanned pregnancy. The possible explanation is women who had an unplanned pregnancy may be too sensitive in terms of confidentiality and privacy due to possible stigma if the pregnancy is out of marriage. Women who had an unplanned pregnancy may also experience greater relationship instability than women whose pregnancies were intended [27]. Another possible explanation is women who had an unplanned pregnancy may request termination of pregnancy, which is not supported by Ethiopian abortion laws.

### 4.1. Limitations of the Study

The presence of high heterogeneity among the included study was sought as a limitation for this study. Absence of data upon requesting the corresponding authors of few articles might affect the results of this study. Due to the absence of primary studies, other regions were not covered in this study. Hence, primary studies aimed to reach those regions are very important.

## 5. Conclusion

Antenatal care service satisfaction is an indicator of the quality of antenatal care. Low satisfaction or dissatisfaction with the antenatal care services hinder women from going to a health facilities to receive prenatal care. Factors such as decreased frequency of visits, long waiting time to see care, provider, privacy, unmaintained, disrespectful treatment, and pregnancy unplanned cause low satisfaction with antenatal care services. In the future, I hope the findings of this research will lead to improvement of the quality of antenatal care services. If we cannot avoid those factors, antenatal care dissatisfaction will make the women flee the health institutions and we will continue to have low antenatal care utilization.

Hence, antenatal care service providers should counsel the women to finish the recommended visits, should treat the mother friendly and respectfully whenever they come for the service. In addition, reproductive health counselling shall be provided to all women of reproductive age to prevent unplanned pregnancy.

## Figures and Tables

**Table 1:** Descriptive summary of thirteen studies included in the meta-analysis of determinants of ANC service satisfaction among women in Ethiopia.

S. no	Authors	Year	Region	Setting	Design	Response rate	Sample size
1	Tesfaye et al.	2017	SNNPR	Health institution	Cross-sectional	100	290
2	Yohanness et al.	2013	SNNPR	Health institution	Cross-sectional	100	363
3	Chemir et al.	2014	Oromia	Health institution	Cross-sectional	100	389
4	Lakew et al.	2018	SNNPR	Health institution	Cross-sectional	100	405
5	Selgado et al.	2019	Oromia	Health institution	Cross-sectional	91	737
6	Asefa et al.	2020	Oromia	Health institution	Cross-sectional	100	358
7	Bekele et al.	2017	Oromia	Health institution	Cross-sectional	95.64	386
8	Mekonnen	2017	SNNPR	Health institution	Cross-sectional	98.8	418
9	Ejigu	2013	Amhara	Health institution	Cross-sectional	93.2	369
10	Kebede	2020	SNNPR	Health institution	Cross-sectional	93	303
11	Muzemil	2014	Addis- Ababa	Health institution	Cross-sectional	100	422
12	Gelaw	2020	SNNPR	Health institution	Cross-sectional	88	340
13	Birhanu et al.	2020	Harari	Health institution	Cross-sectional	97.4	513

## Data Availability

The primary data used to support the findings of this study are available from the corresponding author upon reasonable request.

## Supplementary Materials

Supplementary Materials Additional file 1: a supplementary file. It contains different search strategies used for searching of an articles in databases and gray literature. Additional file 2: report of risk of bias assessment of articles included in the meta-analysis. Additional file 3: report of sensitivity analysis of factors with high heterogeneity .
