# Supplementary material for: Determinants of Antenatal Care Service Satisfaction among Women in Ethiopia: A Systematic Review and Meta-Analysis
Source: Obstet Gynecol Int. 2022 Mar 4;2022:9527576. doi: 10.1155/2022/9527576 (PMC8916880; doi:10.1155/2022/9527576)
Supplement: Supplementary Materials — Additional file 1: a supplementary file. It contains different search strategies used for searching of an articles in databases and gray literature. Additional file 2: report of risk of bias assessment of articles included in the meta-analysis. Additional file 3: report of sensitivity analysis of factors with high heterogeneity . [file 9527576.f1.zip › 9527576.f1/additional file 2.docx]

Table 1: showing sensitivity analysis to identify effect of single study on the pooled results.

| Author name | Criteria  for  inclusion  in the sample clearly defined | Study  subjects  and the  setting  described  in detail | Exposure  Measured in  a valid and reliable way | Objective,  standard  criteria used  for  measurement  of the  condition | Confounding  Factors  identified | Strategies to  deal with  confounding  factors  stated | Outcomes  Measured in  a valid and  reliable way | Appropriate  statistical  analysis  used | Overall  appraisal |
| --- | --- | --- | --- | --- | --- | --- | --- | --- | --- |
| Asefa et al | 0 | 1 | 1 | 1 | 1 | 0 | 1 | 1 | 6/8 |
| Tesfaye et al | 0 | 1 | 1 | 1 | 1 | 0 | 1 | 1 | 6/8 |
| Muzemil | 1 | 1 | 1 | 1 | 1 | 1 | 1 | 1 | 8/8 |
| Kebede et al | 0 | 1 | 1 | 1 | 1 | 1 | 1 | 1 | 7/8 |
| Chemir et al | 1 | 1 | 1 | 1 | 1 | 1 | 1 | 1 | 8/8 |
| Ejigu et al | 1 | 1 | 1 | 1 | 1 | 1 | 1 | 1 | 8/8 |
| Selgado et al | 1 | 1 | 1 | 1 | 1 | 1 | 1 | 1 | 8/8 |
| Bekele et al | 1 | 1 | 1 | 1 | 1 | 1 | 1 | 1 | 8/8 |
| Gelaw et al | 1 | 1 | 1 | 1 | 1 | 1 | 1 | 1 | 8/8 |
| Mekonnen et al | 1 | 1 | 1 | 1 | 1 | 1 | 1 | 1 | 8/8 |
| Birhanu et al | 1 | 1 | 1 | 1 | 1 | 1 | 1 | 1 | 8/8 |
| Lakew et al | 0 | 1 | 0 | 1 | 1 | 1 | 1 | 1 | 6/8 |
| Yohanes et al | 1 | 1 | 1 | 1 | 1 | 1 | 1 | 1 | 7/8 |
