# Supplementary material for: Determinants of Antenatal Care Service Satisfaction among Women in Ethiopia: A Systematic Review and Meta-Analysis
Source: Obstet Gynecol Int. 2022 Mar 4;2022:9527576. doi: 10.1155/2022/9527576 (PMC8916880; doi:10.1155/2022/9527576)
Supplement: Supplementary Materials — Additional file 1: a supplementary file. It contains different search strategies used for searching of an articles in databases and gray literature. Additional file 2: report of risk of bias assessment of articles included in the meta-analysis. Additional file 3: report of sensitivity analysis of factors with high heterogeneity . [file 9527576.f1.zip › 9527576.f1/additional file 3SA. sensitivity analysis.docx]

Table 1: sensitivity analysis of the association of number of antenatal care visit and antenatal care service satisfaction among women in Ethiopia

| Name , year | Pooled odds ratio | 95% CI | I^2^ (%) | p-value |
| --- | --- | --- | --- | --- |
| Yohanness et al , 2013 | 0.66 | 0.42-1.04 | 85.5 | <0.01 |
| Lakew et al , 2018 | 0.60 | 0.36-0.97 | 85.4 | <0.01 |
| Asefa et al, 2020 | 0.63 | 0.38-1.03 | 86.2 | <0.01 |
| Bekele et al, 2017 | 0.60 | 0.37-0.98 | 86.0 | <0.01 |
| Mekonnen, 2017 | 0.63 | 0.39-1.04 | 86.2 | <0.01 |
| Ejigu ,2013 | 0.66 | 0.40-1.07 | 85.3 | <0.01 |
| Muzemil, 2014 | 0.59 | 0.37-0.94 | 85.8 | <0.01 |
| Gelaw, 2020 | 0.54 | 0.37-0.77 | 73.8 | <0.01 |
| Birhanu et al, 2020 | 0.72 | 0.49-1.05 | 73.9 | <0.01 |

Table 2: sensitivity analysis of the association of maternal privacy and antenatal care service satisfaction among women in Ethiopia

| Name , year | Pooled odds ratio | 95% CI | I^2^ (%) | Q | p-value |
| --- | --- | --- | --- | --- | --- |
| Yohanness et al , 2013 | 3.59 | 1.58-8.13 | 93.4 | 30.28 | <0.01 |
| Asefa et al, 2020 | 4.98 | 2.17-11.43 | 91.8 | 24.43 | <0.01 |
| Ejigu ,2013 | 4.45 | 1.60-12.28 | 90.7 | 21.46 | <0.01 |
| **Kebede, 2014** | **2.81** | **1.82-4.34** | **58.8** | **4.85** | **0.09** |

Table 3: sensitivity analysis of the association of respectful treatment and antenatal care service satisfaction among women in Ethiopia

| Name , year | Pooled odds ratio | 95% CI | I^2^ (%) | Q | p-value |
| --- | --- | --- | --- | --- | --- |
| Yohanness et al , 2013 | 4.19 | 1.68-10.47 | 85.4 | 13.7 | <0.01 |
| Selgado et al, 2019 | 4.62 | 1.52-14.36 | 81.0 | 10.55 | <0.01 |
| **Asefa et al, 2020** | **6.99** | **4.98-9.83** | **0.0** | **0.67** | **0.72** |
| Muzemil, 2014 | 4.85 | 1.85-12.71 | 87.4 | 15.92 | <0.01 |

Table 4: sensitivity analysis of the association of maternal place of residence and antenatal care service satisfaction among women in Ethiopia

| Name , year | Pooled odds ratio | 95% CI | I^2^ (%) | Q | p-value |
| --- | --- | --- | --- | --- | --- |
| Yohanness et al , 2013 | 1.41 | 0.48-4.12 | 89.6 | 19.15 | <0.01 |
| Selgado et al, 2019 | 1.09 | 0.46-2.58 | 79.2 | 9.61 | <0.01 |
| Bekele et al, 2017 | 2.05 | 1.29-3.25 | 58.4 | 4.81 | 0.09 |
| Kebede , 2014 | 1.21 | 0.46-3.29 | 90.5 | 20.97 | <0.01 |

Table 5: sensitivity analysis of the association of unplanned pregnancy and antenatal care service satisfaction among women in Ethiopia

| Name , year | Pooled odds ratio | 95% CI | I^2^ (%) | Q | p-value |
| --- | --- | --- | --- | --- | --- |
| Tesfaye et al , 2017 | 0.21 | 0.03-1.31 | 95.9 | 24.37 | <0.01 |
| **Chemir et al, 2014** | **0.50** | **0.38-0.67** | **0.0** | **0.07** | **0.79** |
| Selgado et al, 2019 | 0.19 | 0.03-1.15 | 94.4 | 17.93 | <0.01 |
