# Supplementary material for: Determinants of Antenatal Care Service Satisfaction among Women in Ethiopia: A Systematic Review and Meta-Analysis
Source: Obstet Gynecol Int. 2022 Mar 4;2022:9527576. doi: 10.1155/2022/9527576 (PMC8916880; doi:10.1155/2022/9527576)
Supplement: Supplementary Materials — Additional file 1: a supplementary file. It contains different search strategies used for searching of an articles in databases and gray literature. Additional file 2: report of risk of bias assessment of articles included in the meta-analysis. Additional file 3: report of sensitivity analysis of factors with high heterogeneity . [file 9527576.f1.zip › 9527576.f1/additional file1.docx]

Additional file 1: example of searches for PubMed, Hinari and Google scholar to assess determinants of antenatal care service satisfaction among women in Ethiopia

| Databases | Search terms | Number of studies | Date |
| --- | --- | --- | --- |
| PubMed/MEDLINE | ((((((magnitude[Title]) OR (prevalence[Title])) OR(determinants[Title])) OR ("associated factors"[Title])) AND ("antenatal care"[Title])) OR ("prenatal care"[Title])) AND (satisfaction[Title]) | 42 | 26/6/2020 |
| Hinari | (TitleCombined:(antenatal)) AND ((TitleCombined:("care")) OR (TitleCombined:("prenatal"))) AND (TitleCombined:("care")) AND (TitleCombined:("satisfaction")) | 54 | 12/13/2020 |
| Google scholar | antenatal AND care AND satisfaction | 149 | 12/13/2020 |
| Others (grey literature (electronic library of AAU and Google) |  | 29 | 12/13/2020 |
| Total retrieved articles |  | 274 |  |
